# Early-Life Oxidative Stress and Pediatric Kidney Disease: From Developmental Programming to Preventive Strategies

**DOI:** 10.3390/medicina62101874

**Published:** 2026-09-28

**Authors:** Luca Pecoraro, Alessio Signore, Andrea Dell’Anna, Giulia Carrino, Ilenia Chillura, Flavia Indrio

**Affiliations:** 1 Department of Experimental Medicine, Pediatric Section, University of Salento Hospital “Vito Fazzi”, 73100 Lecce, Italy; 2 Pediatric Unit, Ospedale Vito Fazzi, ASL Lecce, 73100 Lecce, Italy; 3 Pediatric Department, University of Bari Aldo Moro, 70121 Bari, Italy

**Keywords:** oxidative stress, first 1000 days, kidney development, renal programming, DOHaD, nephron endowment, mitochondria, antioxidants, pediatrics

## Abstract

The first 1000 days of life represent a critical window for renal development and long-term kidney health. During this period, oxidative stress and mitochondrial dysfunction may disrupt nephrogenesis, renal maturation, and redox-sensitive developmental pathways, potentially contributing to reduced nephron endowment and greater susceptibility to hypertension and chronic kidney disease later in life. This narrative review examines prenatal, perinatal, and early postnatal factors that may contribute to oxidative renal injury, including maternal metabolic and nutritional conditions, prematurity, impaired fetal growth, perinatal interventions, early-life nutritional and inflammatory factors, and environmental exposures. Particular attention is given to the concept of renal vulnerability and to the distinction between clinical evidence of long-term kidney risk and experimental evidence supporting potential mechanisms of developmental renal programming. Clinical cohort data indicate an increased risk of kidney disease later in life following prematurity and impaired fetal growth, although these associations do not establish oxidative stress as a causal mediator. We also discuss preventive and emerging reprogramming strategies. While mitochondrial-targeted, nutritional, and antioxidant approaches remain promising, current evidence is predominantly preclinical and does not support routine antioxidant supplementation or screening for oxidative stress biomarkers in children. From a practical pediatric perspective, early identification of relevant risk factors, appropriate clinical assessment, optimization of modifiable exposures, and individualized referral to pediatric nephrology represent the most clinically relevant strategies for protecting long-term kidney health.

## 1. Introduction

Over the past few decades, evidence has increasingly suggested that many chronic disorders of adult life may have their origins in early development. In this review, the term “first 1000 days” refers to the period from conception through approximately 24 months after birth. This concept underlies the Developmental Origins of Health and Disease (DOHaD) framework, according to which environmental, metabolic, and nutritional exposures acting during defined developmental windows may induce persistent changes in organ structure and function and shape disease susceptibility across the life course. The kidney is among the organs most susceptible to developmental programming. Renal development begins early in embryonic life and, under physiological conditions, nephrogenesis is completed during the final weeks of gestation, at approximately 34–36 weeks, establishing the nephron endowment that must sustain renal function thereafter. After birth, throughout the postnatal phase of the first 1000 days, the kidney continues to mature functionally through a progressive increase in glomerular filtration, tubular maturation, and hemodynamic adaptation. Disturbances occurring during these critical stages may therefore compromise renal development and function, with long-term consequences. Oxidative stress is one of the major pathogenic mechanisms implicated in renal programming. Under physiological conditions, reactive oxygen and nitrogen species (ROS/RNS) participate in cell signaling, proliferation, differentiation, and organogenesis. When reactive species are produced in excess or antioxidant defenses are insufficient, redox homeostasis is disrupted, leading to damage to lipids, proteins, DNA, and mitochondrial structures. The interaction between ROS and nitric oxide (NO) is especially relevant: an altered ROS/NO balance may reduce NO bioavailability and contribute to endothelial dysfunction and hemodynamic abnormalities involved in the developmental origins of renal and cardiovascular disease [1,2]. A wide range of risk factors may promote oxidative stress during the first 1000 days. These include maternal conditions such as gestational diabetes, infection, exposure to tobacco, alcohol, or other substances, and nutritional imbalance; perinatal events such as prematurity, intrauterine growth restriction, and low birth weight; and postnatal factors including inadequate nutrition, obesity, environmental toxicants, microbiota disruption, and recurrent infections (Figure 1).

Although these early-life exposures differ in nature and timing, they may converge on common pathways characterized by oxidative stress, mitochondrial dysfunction, and altered NO homeostasis. Through epigenetic, inflammatory, and metabolic mechanisms, these changes can interfere with nephrogenesis and subsequent renal maturation, contributing to the process commonly referred to as kidney programming [1,2]. Reduced nephron number is an important structural correlate of adverse developmental exposures [3,4]. Experimental studies support oxidative stress as one mechanism capable of altering nephrogenesis and renal maturation. Because nephron endowment is essentially determined before birth and is not meaningfully regenerated later, a reduced nephron number increases the functional load placed on the remaining nephrons. Compensatory glomerular hyperfiltration and hypertrophy may initially maintain filtration, but over time, they can promote glomerular injury, proteinuria, and higher blood pressure, increasing the likelihood of hypertension and progression to chronic kidney disease (CKD) [3,5,6]. This model integrates the effects of prenatal, perinatal, and postnatal exposures on future kidney risk and identifies opportunities for early-life intervention. The aim of this narrative review is to examine oxidative stress as a major mediator of renal programming during the first 1000 days of life, with particular attention to prenatal, perinatal, and postnatal risk factors, and to potential preventive or therapeutic strategies for reprogramming that target redox balance, the NO system, and mitochondrial function [7,8].

## 2. Materials and Methods

A literature search was conducted in PubMed/MEDLINE for records published or available online from January 1990 through June 2026. The final database search was performed in June 2026. The search strategy combined terms related to kidney development and developmental programming with terms related to oxidative stress and early-life exposures. The principal search strategy was based on combinations of the following terms: (“kidney development” OR “renal development” OR nephrogenesis OR “nephron endowment” OR “renal programming” OR “developmental programming” OR “developmental origins of health and disease” OR DOHaD) AND (“oxidative stress” OR “reactive oxygen species” OR ROS OR “nitric oxide” OR mitochondrial OR mitochondria OR antioxidant OR “redox homeostasis”) AND (prenatal OR fetal OR pregnancy OR maternal OR perinatal OR neonatal OR infancy OR “first 1000 days” OR prematurity OR preterm OR “low birth weight” OR “intrauterine growth restriction” OR IUGR). Studies were considered eligible when they provided information relevant to at least one of the following domains: (i) normal or altered renal development and nephrogenesis; (ii) oxidative or nitrosative stress during prenatal, perinatal, or early postnatal life; (iii) early-life determinants of nephron endowment or subsequent renal vulnerability; (iv) molecular mechanisms potentially linking early-life exposures to renal programming, including mitochondrial dysfunction, nitric oxide signaling, inflammation, epigenetic regulation, and the gut–kidney axis; or (v) preventive or reprogramming strategies potentially relevant to early renal health. Human observational studies, clinical studies, and cohort studies, as well as animal studies, experimental studies, and relevant narrative or systematic reviews, were considered, given the mechanistic and translational scope of the review. Articles were excluded when they were not relevant to kidney development or renal disease, did not address oxidative/redox mechanisms or developmental programming, were published outside the predefined time interval, or did not provide sufficient information relevant to the objectives of the review. Conference abstracts, case reports without mechanistic relevance, editorials, and non-English-language publications were not routinely considered. Relevant publications were selected according to the predefined eligibility criteria based on assessment of titles and abstracts and, when appropriate, review of the full text. Additional relevant publications were identified through reference-list screening of key articles and targeted searches addressing specific mechanistic, clinical, or translational aspects of the review. Figure 1 and Figure 2 were developed with assistance from ChatGPT (OpenAI) to organize and present the concepts defined by the authors. The figures were reviewed and edited by the authors, who take full responsibility for their final content.

## 3. Oxidative Stress as the Potential Driver of Renal Programming

Renal oxidative stress has been proposed as an important mechanistic component of kidney developmental programming during the first 1000 days, based on converging evidence from experimental and translational studies. Oxidative stress occurs when the production of reactive oxygen and nitrogen species (ROS/RNS) exceeds the capacity of endogenous antioxidant systems to neutralize them [7,9]. Experimental and physiological studies have identified the mitochondrial respiratory chain and enzymatic systems such as NADPH oxidases as major sources of renal ROS, with Nox4 being a predominant NADPH oxidase isoform in the kidney. At physiological concentrations, ROS contribute to cell signaling, gene expression, and organogenesis; excessive ROS production, however, can overwhelm antioxidant defenses, including superoxide dismutase, catalase, glutathione peroxidase, and the Nrf2 system, leading to oxidative damage to lipids, proteins, DNA, and mitochondrial structures [9,10]. Experimental studies indicate that the interaction between ROS and nitric oxide (NO) is particularly relevant to renal development. Excess superoxide reacts with NO to form peroxynitrite, reducing NO bioavailability and promoting endothelial nitric oxide synthase (eNOS) uncoupling, which further increases superoxide production [11,12]. Experimental and clinical studies indicate that increased asymmetric dimethylarginine (ADMA), an endogenous inhibitor of nitric oxide synthase, can impair NO signaling; however, whether this pathway directly contributes to developmental renal programming remains incompletely established [8,12]. Experimental studies support a role for ROS/NO imbalance in renal injury and developmental programming, whereas clinical studies provide evidence of its involvement in kidney disease and hypertension; direct human evidence linking this pathway to renal developmental programming remains limited [11,13,14]. Renal development begins early in embryonic life, with nephrogenesis completed at approximately 34–36 weeks of gestation, while functional maturation continues throughout the postnatal phase of the first 1000 days through renal growth, increasing GFR, tubular maturation, and hemodynamic adaptation. Experimental developmental studies indicate that nephron progenitor cells are sensitive to intracellular redox status, which may influence the balance between self-renewal and differentiation [7]. Excessive ROS generation during this period may disturb this balance, accelerate depletion of the progenitor pool, and ultimately reduce nephron endowment [7,15]. In an experimental rat model involving altered signaling through the mitochondrial adaptor p66Shc, increased renal H_2_O_2_ production during nephrogenesis was associated with a reduced glomerular number and greater susceptibility to hypertension and renal injury in adulthood [15]. Experimental and translational studies have identified several mechanisms that may contribute to persistent renal developmental vulnerability, including mitochondrial dysfunction, impaired oxidative phosphorylation, impaired mitochondrial antioxidant defenses such as MnSOD, epigenetic alterations involving DNA methylation, histone modifications and microRNAs, dysregulation of the renin–angiotensin system and nutrient-sensing pathways, and disruption of NOS/NO signaling [8,9,16]. These pathways may therefore contribute to persistent renal developmental vulnerability. The gut–kidney axis, and particularly the gut–redox axis, should be considered a potentially relevant but incompletely established component of developmental renal programming. Direct evidence linking maternal or early-life microbiota alterations to kidney development during the first 1000 days remains limited and should be interpreted cautiously. A recent study combining microbiota analyses from pregnancies complicated by gestational diabetes with complementary in vitro and mouse experiments reported alterations in SCFA availability and GPR43 signaling associated with adverse offspring kidney development [17]. Other animal models of developmental hypertension have shown that maternal dietary or microbiota-targeted interventions can modify offspring gut microbial composition, circulating SCFAs, renal SCFA receptor expression, and later blood pressure [18]. These findings should be viewed as preclinical evidence supporting a possible developmental role for microbiota-derived metabolites rather than as direct evidence of altered nephrogenesis in humans. Evidence concerning SCFA signaling in kidney physiology and development is supported by developmental animal studies and the broader physiological literature [17,18,19]. By contrast, much of the evidence linking dysbiosis, indoxyl sulfate, trimethylamine-N-oxide (TMAO), inflammatory pathways, and oxidative stress to renal injury derives from established CKD or other non-developmental disease settings [20]. Accordingly, mechanisms involving SCFA receptors, TLR4, the aryl hydrocarbon receptor, and Keap1/Nrf2 should currently be regarded mainly as mechanistic hypotheses when extrapolated to renal programming during the first 1000 days. Targeting the gut–redox axis should therefore remain an experimental reprogramming concept pending further developmental and clinical evidence [17,18,20]. Overall, oxidative stress may therefore act as a common mechanistic link between adverse developmental conditions and persistent renal vulnerability.

## 4. Early-Life Exposures Leading to Oxidative Renal Injury

Early-life exposures occurring during the prenatal, perinatal, and early postnatal periods may interact with the immature renal redox system and contribute to oxidative injury and adverse kidney programming. These exposures are particularly relevant when they occur during critical windows of nephrogenesis and renal maturation and involve pathways related to oxidative stress, mitochondrial dysfunction, inflammation, or altered developmental signaling. Maternal metabolic and nutritional conditions, prematurity and perinatal interventions, early-life nutritional and inflammatory factors, and environmental toxicants may therefore influence renal development and long-term kidney vulnerability, although the strength of evidence varies substantially across exposures and is often derived from experimental models [21]. Accordingly, the following sections distinguish, where relevant, between human observational or clinical evidence and mechanistic evidence derived from animal or other experimental models. For several early-life exposures, direct molecular evidence in humans during the first 1000 days is limited or unavailable; in these cases, mechanistic pathways are described based on experimental and translational evidence and should be regarded as proposed rather than established mechanisms. A recent pediatric-focused review from our group provided a broad overview of kidney-health protection during the first 1000 days [21]. The present review has a narrower mechanistic purpose: it focuses specifically on oxidative/nitrosative stress and mitochondrial pathways, explicitly separates human clinical evidence from animal and experimental evidence, and incorporates quantitative long-term kidney-risk estimates where available.

### 4.1. Prenatal Risk Factors

- Gestational diabetes mellitus (GDM): Human observational and experimental studies suggest that intrauterine hyperglycemia is associated with placental mitochondrial dysfunction, impaired oxidative phosphorylation, and increased ROS generation in the setting of altered antioxidant defenses. Experimental and translational evidence suggests that this pro-oxidant environment may induce epigenetic changes, including DNA methylation, histone modifications, and non-coding RNA alterations, that dysregulate genes involved in nephrogenesis, thereby reducing nephron endowment and impairing renal maturation [22,23].
- Maternal tobacco exposure: Experimental mouse studies have shown that maternal cigarette-smoke exposure increases mitochondria-derived ROS in the offspring kidney and is associated with abnormal mitochondrial structure, reduced oxidative-phosphorylation proteins, and lower levels of the mitochondrial antioxidant MnSOD. In these experimental models, renal underdevelopment and albuminuria may follow, while mitochondrial abnormalities can precede albuminuria. Mitochondrial-targeted antioxidant strategies such as L-carnitine and MitoQ attenuated these changes in animal models, supporting a mechanistic role for oxidative injury [16,24,25,26].
- Prenatal alcohol exposure: Evidence derived predominantly from experimental models suggests that ethanol can alter ureteric branching and the expression of key developmental genes, reducing glomerular number through oxidative and apoptotic mechanisms [27].
- Cocaine and other substances of abuse: Experimental and mechanistic evidence suggests that cocaine and other substances of abuse may cause vasoconstriction and uteroplacental hypoxia, increasing oxidative stress and potentially compromising fetal renal vascularization and growth [28]. Direct human evidence for oxidative renal programming associated with these exposures remains limited [28].
- Maternal infections: Experimental and mechanistic evidence suggests that maternal infections may induce systemic inflammatory activation with cytokine and ROS release, potentially creating an inflammatory–oxidative environment that interferes with nephrogenesis [27]. Direct evidence in human pregnancy linking maternal infection to oxidative impairment of nephrogenesis remains limited [27].
- Nutritional deficiencies, particularly iron and zinc: Experimental and translational evidence suggests that deficiencies of zinc, iron, or other key maternal micronutrients may reduce antioxidant capacity, amplify oxidative stress during nephrogenesis, and adversely affect nephron endowment [28]. Direct human evidence linking specific maternal micronutrient deficiencies to oxidative impairment of nephrogenesis remains limited [28].

### 4.2. Perinatal Risk Factors

- Preterm birth: Experimental and translational evidence suggests that delivery before completion of nephrogenesis exposes the immature kidney to a relatively oxygen-rich extrauterine environment and to inflammatory stimuli. This combination may increase oxidative stress and lead to abnormal completion of nephron and podocyte development [27,29]. Long-term clinical cohort data also identify preterm birth as a risk factor for later CKD [30].
- Low birth weight, IUGR, and SGA status: Human observational and clinical evidence identifies these conditions as important correlates of reduced nephron endowment and increased long-term renal vulnerability. Mechanistic evidence, derived largely from experimental and translational studies, suggests that placental insufficiency may produce chronic hypoxia, nitrosative and oxidative stress, impaired ureteric branching, increased global DNA methylation, and caspase-3 activation, leading to apoptosis of the metanephric mesenchyme. Heightened nitrosative stress may also increase susceptibility to tubular injury and tubulointerstitial fibrosis [27,31]. Large-registry data provide direct clinical evidence that low birth weight and SGA are associated with increased risk of later kidney failure [32].
- Nephrotoxic drugs, including aminoglycosides and NSAIDs: Experimental and clinical evidence indicates that aminoglycosides can accumulate in tubular cells and promote ROS generation and mitochondrial injury, whereas NSAID-mediated prostaglandin inhibition may compromise renal hemodynamics in the developing kidney. These mechanisms may increase susceptibility to acute kidney injury, although direct evidence linking early-life nephrotoxic exposure to subsequent CKD through oxidative programming remains limited [29,33].
- Perinatal pharmacological and supportive treatments: The cumulative burden of life-saving neonatal therapies—including nephrotoxic drugs, oxygen supplementation, and mechanical ventilation—may contribute to oxidative stress in the immature kidney. Evidence for this association is derived predominantly from experimental and translational studies [29].
- Mechanical ventilation and oxygen therapy: Experimental and translational studies suggest that relative hyperoxia increases ROS generation in tissues adapted to the fetal environment and may amplify systemic and renal oxidative stress in the immature kidney [29].
- Syndromic disorders, including Down syndrome and DiGeorge syndrome: Clinical and experimental evidence suggests that genetic susceptibility and congenital abnormalities of renal or nephrovascular development may increase renal vulnerability in affected children. Whether these conditions are associated with reduced nephron endowment and oxidative developmental programming remains insufficiently established [29,34].

### 4.3. Postnatal Risk Factors

- Early-life nutrition: Experimental studies indicate that nutritional exposure during the early postnatal period may influence renal development and long-term kidney health. In rodent models, early postnatal overfeeding has been associated with altered postnatal nephrogenesis and with later hypertension, proteinuria, glomerulosclerosis, and renal dysfunction, particularly when rapid postnatal growth follows intrauterine growth restriction [35,36]. In preterm infants, incomplete nephrogenesis at birth may represent an additional period of vulnerability to postnatal nutritional conditions [35,36].
- Overweight and obesity: Pediatric observational studies indicate that obesity is associated with renal functional alterations, including glomerular hyperfiltration, and with cardiometabolic risk factors [37]. Experimental and translational studies further suggest that excess adiposity may promote renal inflammation, activation of NADPH oxidase, increased ROS generation, and oxidative stress [35]. These findings provide a mechanistic context for potential renal vulnerability but do not by themselves demonstrate developmental renal programming during the first 1000 days.
- Recurrent urinary tract infections: Human pediatric studies have shown that recurrent febrile episodes are associated with a higher risk of renal scarring, particularly in children with vesicoureteral reflux or other predisposing abnormalities [38]. This represents clinical evidence of cumulative renal injury rather than direct evidence that recurrent infection causes oxidative developmental programming during the first 1000 days [38].
- Environmental toxicants, including lead and cadmium: Experimental and observational studies have shown that heavy metals such as lead and cadmium can accumulate in renal tissue and are associated with oxidative stress, inflammation, endothelial dysfunction, and tubular and glomerular injury. Experimental evidence further suggests that epigenetic changes may contribute to persistent renal susceptibility; however, direct evidence linking early-life heavy-metal exposure to developmental renal programming remains limited [33].
- Psychosocial stress: Experimental models suggest that chronic psychosocial stress may contribute to hemodynamic disturbances and oxidative programming of hypertension. Direct human evidence linking psychosocial stress during the first 1000 days to renal developmental programming remains limited [35].
- Gut microbiota dysbiosis: Direct evidence that specific microbiota patterns cause adverse renal programming during the first 1000 days remains limited. Experimental data suggest that maternal metabolic or dietary perturbations can alter microbiota-derived SCFAs and renal SCFA-receptor signaling, with effects on offspring kidney development or programmed hypertension [17,18]. In contrast, associations involving specific microbial taxa, protein-bound uremic toxins such as indoxyl sulfate and TMAO, and pathways including TLR4, the aryl hydrocarbon receptor, and Keap1/Nrf2 are derived largely from studies of established kidney disease or broader experimental models [20,39]. These mechanisms should therefore be considered hypothesis-generating rather than direct evidence of developmental renal programming. Early probiotic, prebiotic, and postbiotic approaches should likewise be regarded as experimental reprogramming strategies until specifically validated during the first 1000 days [18,39].

Although the strength and type of evidence vary across exposures, direct molecular evidence in humans is available for only a subset of the associations discussed, whereas many mechanistic links remain supported predominantly by animal, experimental, or translational studies (Table 1).

**Table 1:** Early-life risk factors and predominant oxidative mechanisms potentially contributing to adverse renal programming during the first 1000 days.

Time Window	Stage of Renal Development	Major Risk Factors	Predominant Oxidative Mechanism	Type of Evidence	Ref.
Prenatal (conception to birth)	Onset and completion of nephrogenesis; establishment of nephron number	GDM; tobacco; alcohol; cocaine/other substances; maternal infection; iron/zinc deficiency; placental insufficiency	Placental mitochondrial dysfunction and ROS excess; uteroplacental hypoxia and nitrosative stress; impaired antioxidant defense; epigenetic dysregulation of nephrogenesis	Human Animal Experimental	[16,22,23,27,28]
Perinatal (birth and neonatal period)	Nephrogenesis may be interrupted in preterm infants; immature kidney exposed to extrauterine conditions	Prematurity; low birth weight; IUGR/SGA; nephrotoxic drugs; oxygen/ventilation; syndromic disorders	Relative hyperoxia and ROS bursts; tubular mitochondrial injury; inflammatory–oxidative stress; apoptosis of metanephric mesenchyme	Human Animal Experimental	[27,29,30,31,32,33,34]
Postnatal (within the first 1000 days)	Functional maturation: renal growth, rising GFR, tubular maturation, hemodynamic adaptation	Inadequate nutrition/overfeeding; obesity; recurrent UTI/reflux nephropathy; heavy metals; psychosocial stress; dysbiosis	Chronic inflammation and NADPH oxidase activation; infection-related oxidative bursts; metal accumulation; altered gut–redox axis with ↓ SCFAs and ↑ uremic toxins	Human Animal Experimental	[17,18,20,33,35,36,37,38,39]

## 5. Pediatric Implications of Early-Life Renal Vulnerability

Evidence from large population-based studies directly supports an association between selected adverse early-life exposures and later kidney disease, particularly prematurity and impaired fetal growth. In a Swedish national cohort of 4,186,615 singleton births, preterm birth (<37 weeks) was associated with an increased risk of CKD from childhood to mid-adulthood (aHR 1.94, 95% CI 1.74–2.16), while extremely preterm birth (<28 weeks) was associated with a higher risk (aHR 3.01, 95% CI 1.67–5.45). The association was strongest at ages 0–9 years (aHR 5.09, 95% CI 4.11–6.31). Importantly, CKD was diagnosed in 4305 individuals (0.1%) during follow-up, indicating that the absolute risk remained low despite the increased relative risk associated with preterm birth [30]. In a Norwegian registry cohort of 2,679,967 individuals, low birth weight was associated with an increased risk of ESRD (aHR 1.61, 95% CI 1.38–1.98), while SGA was also associated with increased risk (aHR 1.44, 95% CI 1.22–1.70) [32]. These findings provide clinically relevant evidence of increased long-term renal risk following prematurity and impaired fetal growth. However, they do not establish oxidative stress as the causal mediator of this risk. Evidence from established kidney disease and experimental models remains useful for generating mechanistic hypotheses but should be clearly distinguished from clinical evidence of developmental renal programming. From a pediatric practice perspective, maternal and pregnancy-related factors, neonatal and perinatal factors, and early postnatal factors can be considered contextual indicators of renal vulnerability. Maternal and pregnancy-related factors include maternal hypertension or preeclampsia, gestational diabetes, maternal infections, maternal smoking, and other significant maternal conditions or exposures. Neonatal and perinatal factors include prematurity, IUGR/SGA or low birth weight, perinatal complications, and congenital anomalies of the kidney and urinary tract (CAKUT) or a solitary kidney. Early postnatal factors include recurrent febrile urinary tract infections, obesity or excessive weight gain, psychosocial stress, and other relevant early-life exposures. These factors should be interpreted in the context of the overall clinical profile rather than as automatic referral criteria. Current evidence does not support screening children using unvalidated oxidative-stress biomarkers or referring them to pediatric nephrology solely on the basis of a single historical risk factor. Clinical assessment should instead rely on established measures, such as blood pressure; urinalysis for proteinuria or albuminuria, when indicated; serum creatinine/eGFR, when indicated; and renal imaging, when appropriate for structural indications. Referral to pediatric nephrology should be considered when a combination of relevant risk factors and/or clinical findings is present, according to the overall clinical profile.

## 6. Potential Reprogramming Strategies

Because oxidative stress and mitochondrial dysfunction may contribute to adverse renal programming during the first 1000 days, interventions aimed at preserving redox homeostasis and mitochondrial function have been proposed as potential strategies for reprogramming. Importantly, reprogramming should be distinguished from routine antioxidant treatment. At present, prevention of modifiable maternal, perinatal, nutritional, and environmental insults remains the most clinically relevant approach, whereas nutritional, antioxidant, pharmacological, and nutraceutical interventions aimed at renal reprogramming remain experimental and lack established clinical evidence during the first 1000 days of life. Studies of established kidney disease have implicated alterations in mitochondrial number, distribution, structure, and function in kidney disease progression [40]. These findings provide a mechanistic context for, but not direct evidence of, mitochondrial involvement in developmental renal programming. Experimental strategies include mitochondria-targeted antioxidants, modulation of mitochondrial permeability and fission, stimulation of mitochondrial biogenesis, and approaches targeting microRNAs and other regulators of mitochondrial homeostasis [41,42]. Experimental studies have identified ferroptosis, an iron-dependent form of regulated cell death characterized by lipid peroxidation, as a potential target because of its interaction with oxidative stress, mitochondrial dysfunction, and iron metabolism [41]. Mitochondria-targeted interventions have shown protective effects in experimental models of ischemia–reperfusion injury and AKI-to-CKD progression, particularly by limiting mitochondrial ROS production and tubular injury [42,43]. However, these findings remain predominantly preclinical and cannot yet be extrapolated to healthy children or to the developmental period. Similarly, evidence from clinical trials conducted in adults with established CKD cannot be directly extrapolated to fetal or neonatal nephrogenesis, as these populations differ fundamentally in developmental stage, renal biology, treatment targets, and clinical context. Nutritional optimization represents a more clinically relevant component of early prevention. Zinc contributes to antioxidant defense through its role in copper–zinc superoxide dismutase, and maternal zinc restriction has been associated in animal models with lower birth weight, vascular alterations, and increased susceptibility to hypertension in adulthood, with potential implications for nephrogenesis [44]. Selenium is also involved in antioxidant protection and redox regulation. Although low selenium concentrations have been reported in patients with AKI and CKD, these observations reflect established kidney disease rather than developmental programming, and supplementation studies remain limited and heterogeneous [45]. Magnesium participates in multiple metabolic pathways. Observational studies in patients with established CKD have associated lower magnesium concentrations with CKD progression and cardiovascular complications; however, these findings do not constitute evidence of developmental renal reprogramming, and randomized trials remain insufficient to support systematic supplementation [46]. Thus, these findings support adequate maternal and early-life nutrition rather than routine antioxidant supplementation. Bioactive compounds have also attracted interest because of their potential effects on mitochondrial function, oxidative stress, and inflammation. Polyphenols such as resveratrol, quercetin, catechins, and curcumin have been investigated mainly in experimental models, with particular interest in SIRT1 and PGC-1α signaling and mitochondrial biogenesis [40]. Overall, mitochondria-targeted interventions, micronutrients, polyphenols, and other bioactive compounds represent potential candidates for investigation in future renal reprogramming studies. However, most evidence derives from animal models or from patients with established kidney disease rather than from pregnancy, infancy, or early childhood. A reduction in oxidative stress, therefore, does not necessarily demonstrate an increase in nephron endowment, preservation of renal reserve, or prevention of CKD later in life. Future studies should specifically address the developmental window in which renal programming occurs and determine whether targeted interventions can modify mitochondrial function, renal development, and long-term renal outcomes. At present, reprogramming remains a promising research concept rather than an established therapeutic strategy, while prevention of adverse early-life exposures and optimization of maternal and infant health remain the primary clinical priorities.

## 7. Conclusions

Current evidence supports the first 1000 days of life as a biologically critical window for future kidney health. During this period, essential processes of nephrogenesis and renal maturation take place, making the developing kidney particularly vulnerable to metabolic, nutritional, inflammatory, and environmental insults. Oxidative stress may represent a common pathway linking these early-life exposures to adverse renal programming through impaired NO bioavailability, mitochondrial dysfunction, inflammation, and persistent epigenetic changes. This concept has practical implications for pediatric care. Maternal and pregnancy-related factors, including hypertension or preeclampsia, gestational diabetes, maternal infections, smoking, and other significant maternal conditions or exposures; neonatal and perinatal factors, including prematurity, IUGR/SGA or low birth weight, perinatal complications, and CAKUT or a solitary kidney; and early postnatal factors, including recurrent febrile urinary tract infections, obesity or excessive weight gain, psychosocial stress, and other relevant early-life exposures, should be considered when assessing renal vulnerability. These factors are not automatic referral criteria and should be interpreted together with the overall clinical profile. When a combination of relevant risk factors and/or clinical findings is present, referral to pediatric nephrology may be considered. In children without relevant risk factors or clinical findings, routine follow-up by the primary care pediatrician remains appropriate, with monitoring of growth and blood pressure, promotion of a healthy lifestyle, parental education, and reassessment of risk factors during subsequent visits. When pediatric nephrology assessment is indicated, surveillance may include individualized assessment of the overall risk profile, monitoring of growth, blood pressure, and urinalysis when appropriate, assessment of renal function over time, renal ultrasound when clinically indicated to evaluate kidney size and structure, and early identification and management of modifiable cardiovascular and renal risk factors. From a preventive perspective, the priority remains reducing modifiable early-life insults and promoting optimal maternal and infant health. Adequate nutrition, prevention of excessive weight gain, avoidance of unnecessary nephrotoxic or environmental exposures, and support of a healthy gut microbial environment represent practical measures that may contribute to a healthier renal trajectory. Although modulation of oxidative stress through micronutrients, polyphenols, mitochondria-targeted compounds, and other bioactive interventions is an emerging area of research, current evidence is insufficient to support routine antioxidant supplementation or unvalidated screening of oxidative-stress biomarkers for renal protection in children. Most available data remain preclinical or derive from populations with established kidney disease, and evidence that an intervention reduces oxidative stress does not necessarily demonstrate that it can modify nephron endowment or prevent CKD. The clinical framework proposed in Figure 2 reflects expert opinion and is intended to provide a practical approach to integrating early-life risk information with conventional clinical assessment; it is not a validated screening or referral guideline.

**Figure 2:**
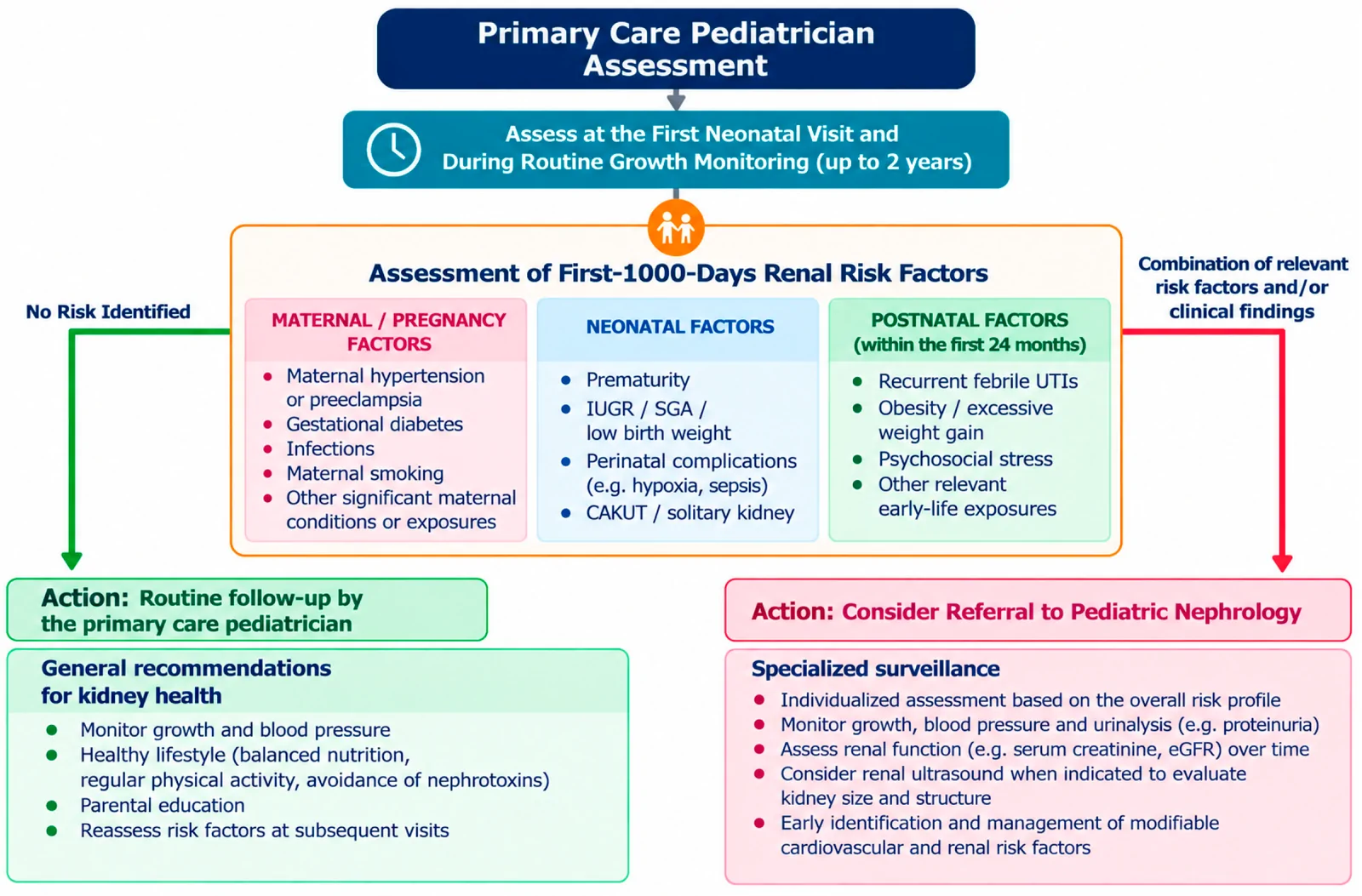
Expert-opinion pediatric framework for integrating early-life renal risk information with conventional clinical assessment.

Future research should therefore focus on the developmental window during which renal programming occurs and determine whether targeted interventions can effectively preserve renal reserve and improve long-term outcomes. Antioxidant and mitochondrial strategies remain promising areas of research, but their clinical role in pediatric renal prevention remains unproven. Current practice should therefore prioritize validated clinical assessment, reduction in modifiable risk factors, and individualized specialist involvement when conventional kidney abnormalities or a combination of relevant risk factors and/or clinical findings are present.

## Figures and Tables

**Figure 1:**
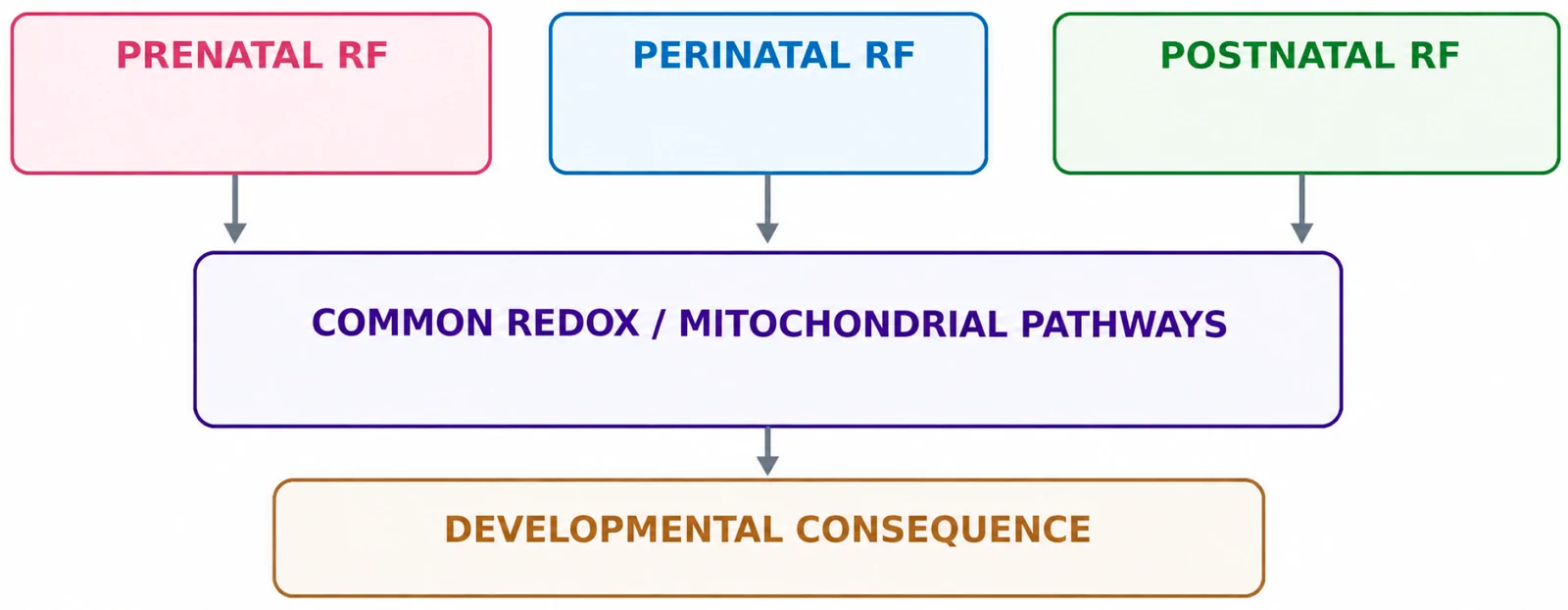
Conceptual pathway linking early-life exposure windows to oxidative/redox mechanisms, altered renal development, and long-term renal vulnerability. RFs: risk factors.

## Data Availability

No new data were created or analyzed in this study. Data sharing is not applicable to this article.

## Abbreviations

The following abbreviations are used in this manuscript:

AKI	Acute Kidney Injury
ADMA	Asymmetric Dimethylarginine
aHR	Adjusted Hazard Ratio
CAKUT	Congenital Anomalies of the Kidney and Urinary Tract
CI	Confidence Interval
CKD	Chronic Kidney Disease
DOHaD	Developmental Origins of Health and Disease
eNOS	Endothelial Nitric Oxide Synthase
ESRD	End-Stage Renal Disease
FSGS	Focal Segmental Glomerulosclerosis
GDM	Gestational Diabetes Mellitus
GFR	Glomerular Filtration Rate
GPR43	G Protein-Coupled Receptor 43
IUGR	Intrauterine Growth Restriction
MnSOD	Manganese Superoxide Dismutase
NADPH	Nicotinamide Adenine Dinucleotide Phosphate
Nox4	NADPH Oxidase 4
NO	Nitric Oxide
NOS	Nitric Oxide Synthase
NOX	NADPH Oxidase
Nrf2	Nuclear Factor Erythroid 2–Related Factor 2
NSAIDs	Nonsteroidal Anti-Inflammatory Drugs
PGC-1α	Peroxisome Proliferator-Activated Receptor Gamma Coactivator 1-Alpha
ROS	Reactive Oxygen Species
RNS	Reactive Nitrogen Species
SCFA	Short-Chain Fatty Acid
SGA	Small for Gestational Age
SIRT1	Sirtuin 1
TLR4	Toll-Like Receptor 4
TMAO	Trimethylamine N-Oxide
UTI	Urinary Tract Infection
